# Introduction of invasive mosquito species into Europe and prospects for arbovirus transmission and vector control in an era of globalization

**DOI:** 10.1186/s40249-023-01167-z

**Published:** 2023-11-30

**Authors:** Renke Lühken, Norbert Brattig, Norbert Becker

**Affiliations:** 1 Bernhard Nocht Institute for Tropical Medicine, 20359 Hamburg, Germany; 2 Institute for Dipterology, 67346 Speyer, Germany; 3 Institute for Organismal Studies (COS), University of Heidelberg, 69117 Heidelberg, Germany

**Keywords:** Invasive mosquito, Spread, Outbreak, Mosquito-borne virus, Asian tiger mosquito, Arbovirus, Control strategy, Globalization Europe

## Abstract

**Background:**

Mosquito research in Europe has a long history, primarily focused on malaria vectors. In recent years, invasive mosquito species like the Asian tiger mosquito (*Aedes albopictus*) and the spread of arboviruses like dengue virus, chikungunya virus or bluetongue virus have led to an intensification of research and monitoring in Europe. The risk of further dissemination of exotic species and mosquito-borne pathogens is expected to increase with ongoing globalization, human mobility, transport geography, and climate warming. Researchers have conducted various studies to understand the ecology, biology, and effective control strategies of mosquitoes and associated pathogens.

**Main body:**

Three invasive mosquito species are established in Europe: Asian tiger mosquito (*Aedes albopictus*), Japanese bush mosquito (*Ae. japonicus*), and Korean bush mosquito (*Aedes koreicus*). *Ae. albopictus* is the most invasive species and has been established in Europe since 1990. Over the past two decades, there has been an increasing number of outbreaks of infections by mosquito-borne viruses in particular chikungunya virus, dengue virus or Zika virus in Europe primary driven by *Ae. albopictus*. At the same time, climate change with rising temperatures results in increasing threat of invasive mosquito-borne viruses, in particular Usutu virus and West Nile virus transmitted by native *Culex* mosquito species. Effective mosquito control programs require a high level of community participation, going along with comprehensive information campaigns, to ensure source reduction and successful control. Control strategies for container breeding mosquitoes like *Ae. albopictus* or *Culex* species involve community participation, door-to-door control activities in private areas. Further measures can involve integration of sterile insect techniques, applying indigenous copepods, *Wolbachia* sp. bacteria, or genetically modified mosquitoes, which is very unlike to be practiced as standard method in the near future.

**Conclusions:**

Climate change and globalization resulting in the increased establishment of invasive mosquitoes in particular of the Asian tiger mosquito *Ae. albopictus* in Europe within the last 30 years and increasing outbreaks of infections by mosquito-borne viruses warrants intensification of research and monitoring. Further, effective future mosquito control programs require increase in intense community and private participation, applying physical, chemical, biological, and genetical control activities.

## Background

Mosquitoes as hematophagous ectoparasites play an important role as pest species and vectors of pathogens [1]. Therefore, research on mosquitoes has a long history worldwide. This is especially true for tropical regions with a high burden of vector-borne diseases. Malaria parasites and the dengue virus (DENV) are the most important pathogens at the global scale with a total of more than 500,000 human deaths per year [2]. Europe also has a long history of research on the ecology of mosquitoes and their associated pathogens [3]. However, most research until the twenty-first century was driven by the interest in the ecology and spatial–temporal distribution of the malaria vectors as several countries had ongoing circulation of different *Plasmodium* species.

Intensive studies were conducted at the beginning of the twentieth century especially in European areas with a burden of malaria cases. However, the disease disappeared by vector control, drainage measurements, drugs and general improvement of the hygienic status. Thus, the interest in mosquitoes as an object of research significantly had declined in most European countries [4].

Over the last decades research on mosquitoes in Europe and systematic mosquito control programs were predominantly restricted to areas with a high burden of mosquito plagues. This situation changed by two important events: the spread of invasive mosquito species since 1990 especially of the Asian tiger mosquito *Aedes albopictus* and the emergence of vector-borne pathogens, e.g. the arthropod-borne (arbo) viruses chikungunya virus (CHIKV), transmitted by the *Ae. albopictus* and of bluetongue virus, transmitted by biting midges (*Culicoides* spp.) [5]. Before the outbreaks of bluetongue virus in Central Europe the risk for the emergence of arboviruses north of the Alps was very low. Especially because the main vectors for arboviruses were regarded to be missing in Germany, e.g., *Culicoides imicola* for bluetongue virus or *Ae. albopictus* for CHIKV. However, the outbreak of bluetongue virus in North-West Europe in 2006 [6] and later followed by Schmallenberg virus in 2011 [7] demonstrated the potential of arbovirus transmission in Central Europe by native hematophagous insects. At the same time the first eggs of the Asian tiger mosquito were detected at a highway station in South-West Germany [8]. The mosquito species is known to be a highly invasive species with a high vector capacity for several important pathogens, as DENV or CHIKV. Both, the bluetongue virus outbreaks and detection of *Ae. albopictus* eggs, demonstrated the huge lack of knowledge regarding native and potentially invasive arthropod vector species in Central Europe including the lack of entomological trained professionals.

The outbreak of bluetongue virus and detection of exotic mosquito species resulted in the intensification of the research on mosquito-borne diseases North of the Alps, collecting baseline information on the ecology of mosquitoes and spatial–temporal distribution of vectors and associated pathogens. Intensified monitoring led to the detection of the mentioned exotic mosquitoes and the ongoing circulation of several arboviruses and parasites like *Dirofilaria* [9–12]. The risk for the further spread of exotic species and ongoing circulation of mosquito-borne pathogens have been expected in face of ongoing globalization and climate warming.

### Current status of invasive mosquito species in Europe

Different exotic mosquito species are regularly introduced to Europe [13]. However, only three different exotic mosquito species are established in Europe: the Asian tiger mosquito (*Ae. albopictus*), the Japanese bush mosquito (*Ae. japonicus*) and the Korean bush mosquito (*Ae. koreicus*) [14]. The high invasive capacity of these *Aedes* species is driven by several ecological traits, but most importantly drought-resistant eggs and the ability to lay hibernating eggs. Thereby, the dispersal of the exotic mosquito species on different scales, i.e., continental, national, regional, is driven by different modes of human mobility dynamics. Spread between continents is predominantly the result of the transport of eggs attached to different goods with plants (e.g. lucky bamboo) and tires by freights on ships with the highest relevance [15]. Aircrafts play a relevant role in the spread [13]. On the national and regional scale, however, transport via cars and trains are the most important modes of dispersal [8, 14]. Due to the relatively small flight range of invasive species [16], active dispersal of the mosquito species probably only plays an underneath role on the local scale.

*Aedes albopictus* is one of the most invasive species in the world. Originating from the Asian region, the species spread all over the world. In Europe, the species was first established in Italy in 1990 and since then spreading widely around the Mediterranean Sea and towards Central Europe [5, 14, 17]. First detected north of the Alps in Germany in 2007 [5], subsequent monitoring activities confirmed regular introductions via human-mediated transport of adult mosquitoes along the highways and several established populations especially in southern Germany. At least since 2016 the species must be considered established with overwintering populations north of the Alps [18] with the recently established most northern population for Europe in Berlin, Germany (personal observation).

The second exotic species is *Ae. japonicus*. The species is considered established in Belgium since 2002 and is especially widely distributed in Central Europe [19, 20]. Populations of the Japanese bush mosquito have already been detected in more than 10 countries [21]. In 2008, the first invasive spread of this species in Europe was reported in Switzerland. Since then, it has even become there a dominant mosquito species. In order to prevent further spread within the country, the Netherlands and Belgium conducted systematic control measurements [22–24]. Shortly after its introduction in Switzerland, populations were established at several sites in southern Germany [23]. Since the first detection, the species spread over wide parts of Germany [14, 21] from South-West Germany up to the coast (personal observation).

The third exotic species is the Korean bush mosquito (*Ae. koreicus*). The species is established with relatively small populations in several countries in Europe with a main focus in Central Europe, e.g. Italy, Belgium, Slovenia, Hungary or the Netherlands [25–28]. In Germany there is currently one stable, small population known in Wiesbaden, southern Germany [29].

### Mosquito-borne viruses in Europe

**Fig. 1:**
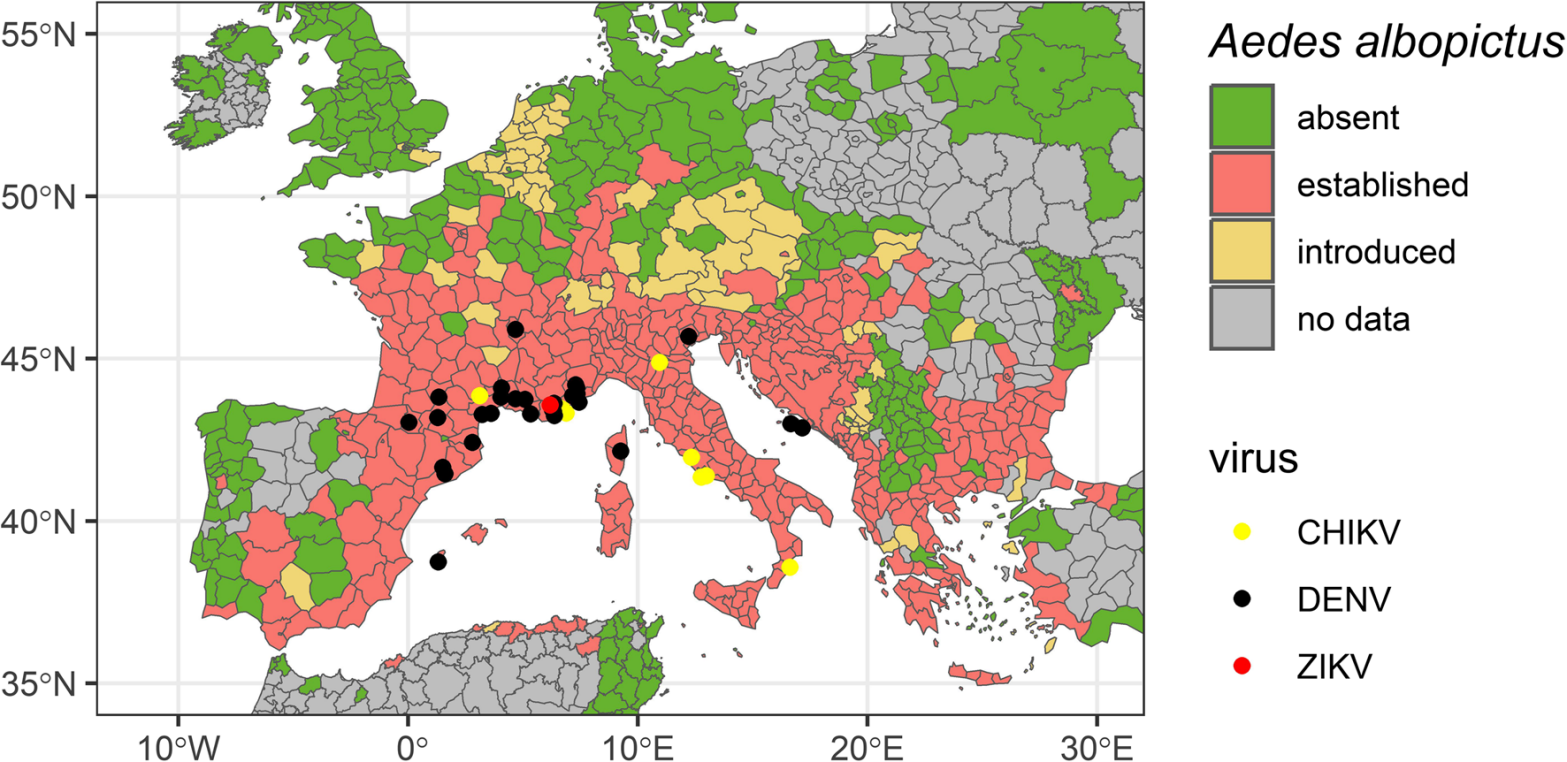
Invasion status for *Aedes albopictus* in Europe and local outbreaks of chikungunya virus (CHIKV), dengue virus (DENV) and Zika virus (ZIKV) (data source: https://www.ecdc.europa.eu/en)

Over the last two decades of vector invasion, we observed increasing numbers of outbreaks of mosquito-borne viruses in Europe. Thereby we can distinguish two different drivers. Firstly, the establishment of *Ae. albopictus* in wide parts of Southern Europe allow the circulation of exotic viruses like DENV [24, 25], Zika virus (ZIKV) [26] or chikungunya virus (CHIKV) (Fig. 1). This process must be separated from the increasing activity of mosquito-borne viruses transmitted by our native *Culex* mosquito species, especially Usutu virus (USUV) and West Nile virus (WNV). It is highly likely that the circulation of DENV, ZIKV and CHIKV only became possible due to the establishment of *Ae. albopictus*. Native mosquito taxa probably only have low or no vector capacity for these viruses. In contrast, USUV and WNV are transmitted by our native *Culex* species. The three exotic mosquito species *Ae. albopictus*, *Ae. japonicus* and *Ae. koreicus* probably are no good vectors for both viruses [30–32].

The global spread of *Ae. albopictus* is a significant public health concern due to its aggressive daytime-biting behaviour and its ability to feed on both humans and animals, making it a potential bridge vector for zoonotic pathogens [33, 34] (Fig. 1). This mosquito species is a known vector of various emerging arboviruses, such as DENV, CHIKV, and ZIKV [33, 35, 36]. The spread of *Ae. albopictus* in Europe has led to the repeated occurrence of autochthonous CHIKV and DENV infections. For instance, in 2007, a CHIKV epidemic occurred in Italy due to a traveller from India, resulting in over two hundred clinical cases [37]. Since then, small-scale circulation has been observed in France in 2010, 2014, and 2017, and a major CHIKV outbreak occurred in Italy in 2017 [38–40]. Additionally, several autochthonous DENV infections have been reported in recent years in France and Croatia [41–45]. Furthermore, the first observation of autochthonous ZIKV infections in France was reported [46, 47]. Laboratory studies using *Ae. albopictus* populations from Germany and Italy have demonstrated their vector competence for ZIKV and CHIKV [48–51].

The Asian bush mosquito (*Ae. japonicus*) is widespread in Central Europe and can have very high population densities. The species is well adapted to temperature climatic conditions with a long seasonal activity also at lower temperatures compared to *Ae. albopictus* [52]. The species probably has a broad host-feeding patterns with a preference for humans, but also non-human mammals or birds [53, 54]. This makes the species a potential vector of zoonotic arboviruses. Vector competence studies proved *Ae. japonicus* as a potential vector for different viruses, including the Japanese Encephalitis virus, WNV, and Saint Louis encephalitis virus [30, 55, 56], but potentially also for the La Crosse virus and CHIKV [57, 58].

The closely related mosquito species *Ae. koreicus* is considered to have a similar ecology as *Ae. japonicus*. Experimental studies prove a vector competence for CHIKV, ZIKV and also the filarial parasite *Dirofilaria immitis* [32, 59, 60]. However, the species relevance as a vector in the field is unknown but is considered to play a role as a vector of the Japanese encephalitis virus in its area of origin [61].

In summary, vector competence studies with European populations of *Ae. japonicus* and *Ae. koreicus* have demonstrated that the species probably only have a very low vector competence for currently circulating pathogens in Europe or only at high temperatures [30–32]. The populations *Ae. albopictus* are probably too small, spatially restricted and the temperatures are still too low to allow transmission of exotic viruses north of the Alps.

In contrast to the future risk of arbovirus transmission by invasive mosquito species, there is already ongoing circulation of several arboviruses through native species. Three viruses can be highlighted here: Sindbis virus, USUV and WNV. Large outbreaks with many human cases are only observed in northern Europe [62]. In contrast, USUV and WNV circulate in South, South-Eastern and Central Europe [63, 64]. All three pathogens have similar transmission cycles including an enzootic cycle between *Culex* mosquitoes and birds.

Since the start of intensified monitoring of mosquito-borne pathogens in 2009, different arboviruses and *Dirofilaria* spp. parasites have been detected in native mosquito species in Germany. Sindbis virus is regularly detected in mosquitoes and birds [65–68]. Several mosquito species in Germany have a very high vector competence for Sindbis virus [69]. However, compared to Finland or Sweden for unknown reasons huge outbreaks with clinically sick humans are not observed. The Batai virus is regularly observed in vertebrates in Germany with high seroprevalence in sheep, goats, and cattle, but clinical signs of Batai virus infections are rarely detected [70, 71]. The same applies to *Dirofilaria repens* and *D. immitis*, which probably also circulates continuously in Germany [9–11]. However, so far only few autochthonous human cases were detected in eastern Germany [9].

USUV and WNV show a continuous, yearly circulation after the respective first detection in 2018 [63, 64]. Both viruses share a similar transmission cycle with birds and amplification hosts and different *Culex* taxa as main vector species. The viruses can overwinter in the female adult mosquitoes [72, personal observation], which probably is the main factor allowing long-term local establishment although not every year outbreaks of the virus are observed. Both viruses are pathogenic for humans. However, USUV only rarely causes severe disease [73], while WNV is a serious risk for humans and equines [63].

USUV was first virus discovered in Europe in 1996, after causing deaths among Eurasian blackbirds (*Turdus merula*) in Italy [74]. Since then, it has spread to other countries, including Austria, Belgium, Czech Republic, France, Germany, Hungary, Spain, Switzerland, and the Netherlands [75–79]. The virus has caused outbreaks among wild and/or captive birds, often resulting in a significant die-off of blackbirds and captive great grey owls (*Strix nebulosa*) [76]. The emergence of USUV has posed a significant challenge for wildlife management authorities in affected regions. As a result, there has been a growing effort to monitor and study the virus, with the aim of developing effective prevention and control strategies. USUV was first detected in a mosquito pool in 2010 in Southwest Germany [80]. Only one year later, the first bird die-off was observed in 2011/12 [78]. Over the following years, USUV spread over large areas of Germany causing massive die-offs of birds and since 2018 have to be considered to spread all over Germany [64, 77, 81, 82]. The virus is known to result in significant decline of the population in European blackbirds [81]. USUV regularly infects humans, but the infections probably do not cause significant health risk for humans except for immunosuppressed patients [73].

WNV is a widely distributed mosquito-borne virus in Europe, with particular focus on South-Eastern Europe and Italy [46]. However, low levels of WNV activity have also been reported in neighbouring countries such as Germany, France, Austria, and the Czech Republic. In light of this, numerous monitoring programs have been implemented in Germany over the past ten years to screen for WNV RNA and antibodies against WNV in birds, horses, mosquitoes, and chicken eggs [67, 82–85]. The first epizootic emergence of WNV was only observed in Germany in 2018 [86]. The virus shows significant circulation especially in Central-Eastern Germany. Since 2018, yearly circulation is observed with infections in birds and horses but also infection of humans occurred every year and one fatal case was reported in 2020 [63]. WNV is also transmitted by our native mosquito species [87]. We know that different *Culex* species are important vectors, while *Cx. torrentium* must be considered as one of the most important vector species with very high transmission rates [88].

Increasing temperatures in the course of climate warming will significantly change the risk of transmission for vector-borne pathogens in Europe [89]. Increasing temperatures will result in a further spread of the thermophilic Asian tiger mosquito [90]. Over the last years we have seen a tremendous increase in the detection of different mosquito populations at different sites north of the Alps [18]. The highest risk for the future must be expected for outbreaks with CHIKV. The virus can also be transmitted at relatively low temperatures, thus, the spatial risk in Europe currently is probably predominantly restricted by the further spread and increasing population size of *Ae. albopictus* [50]. However, local circulation of other exotic pathogens like DENV or ZIKV, which are already observed in Mediterranean areas can be expected in the course of the spread of the *Ae. albopictus* in combination with increasing temperatures.

Nevertheless, the prediction of the impact of climate warming on these pathogens is a complex issue that requires more research. We know that the risk of transmission is strongly affected by the extrinsic incubation period (EIP), which represents the time period between the uptake of a pathogen through a vector and the re-transmission after development and replication in the vector. For most pathogens, the EIP is directly temperature-dependent, with increase of the temperatures the risk of transmission also increases. This has been experimentally demonstrated for the most important pathogens circulating in Europe, such as ZIKV [49] or WNV [88]. There are, however, exceptions to this rule, such as CHIKV, which does not seem to show a clear correlation between EIP and temperature [30].

The unambiguous temperature dependence of the transmission of WNV by *Culex* mosquitoes connotes that warmer temperatures are required for approximately two weeks with significant temperatures of over 20 ℃ [88]. This explains the main distribution of WNV in South-Eastern Europe and the first observed larger outbreak of West Nile fever in Germany during the heat summer in 2018 [63]. This definitive temperature dependence is also observed for the small outbreaks of ZIKV and DENV in the Mediterranean region [41–47]. Thus, both viruses require relative high temperature for successful transmission [49]. However, while we have a good understanding of the impact of climate warming on the development of pathogens in mosquitoes [31, 49, 88] and the ecology of exotic mosquito species like *Ae. albopictus* [90], there is still a great lack of knowledge regarding the impact of climate warming on native mosquito species. This is due in part to the still low research focus on these species, resulting in a lack of systematic time series data. Further research is needed to better understand the impact of climate warming on these species and the potential consequences for public health.

### Control of mosquitoes with special regard to invasive mosquitoes

With the disappearance of malaria on the European continent in the 1950s the country-wide activities were strongly reduced. Laws which regulated mosquito control on a state level were based on medical aspects only. These laws were cancelled in many states as Germany since mosquitoes were no longer considered as a medical threat to humans.

The reasons for the decline of malaria in Europe are multifold:

- First, the appearance of quinine—extracted from the bark of the cinchona tree—as a treatment for malaria was introduced by Pierre Pelletier in 1820 and the consistent treatment of malaria patients led to a continual reduction in the numbers of infected people. This in turn led to a drastic decrease in the probability of a mosquito taking up the parasite as *Plasmodium* spp. through human blood meals and becomes infected.
- Ground water levels have steadily become lower through the canalization of rivers reducing the development of a large mosquito population. A reduction in malaria infection was achieved in Europe through hydraulic engineering measures before the developmental cycle of the disease-causing agent was known.
- Lifestyle changes in humans have resulted in reduced contact with *Anopheles* mosquitoes. For example, stables and living quarters, once together in one complex, are now separate. The female *An. maculipennis* s.l. are mainly zoophilic, meaning they prefer large mammals as hosts for their blood meals, such as cows and horses, thereby acting as vectors.
- Central Europe was climatically borderline for malaria parasites. This is one basic reason why eradication was possible. Today, all the malaria vector-competent mosquitoes are still present in Germany, however, indigenous malaria has disappeared in Central Europe, excluding single conjectural cases. This is also the case in Southern Europe, even with its more favourable climate [89]. Nevertheless, in recent years again autochthonous cases are reported from Southern Europe [46].

The high living and medical standards in Europe will avoid an epidemic re-emergence of malaria. New cases as they occur regularly in Southern Europe will be immediately treated and thus the *Plasmodium*-mosquito contact will be eliminated [89].

In general, the biology of the target mosquito, its importance as nuisance and vector species decides which control strategy must be chosen. In Europe we can differ in general between.

- the control of mosquitoes in wetlands which hatch in masses after flooding and have a great ability for dispersal after emerging such as floodwater mosquitoes *(Ae. vexans*, *Ae. sticticus* or *Ae. caspius*).
- the control of container-breeding mosquitoes and human-made habitats in urban and suburban areas. These species usually have a limited flight range like *Ae. albopictus, Cx. pipiens* or *An. plumbeus*.

As a principle, it is advisable to control immature stages because they are as populations usually defined and concentrated in their breeding sites, whereas adults spread from their breeding grounds several hundred meters (e.g., mosquitoes breeding in urban areas like *Cx. pipiens* or *Ae. albopictus*) up to several kilometres like the floodwater mosquitoes *Aedes vexans* or *Ae. sticticus* [16, 91]. However, when transmission of pathogens takes place, adults as a source for infections have to be controlled at least 100 m around the infection sites in the urban areas. The establishment of new populations of *Ae. albopictus* is mainly based on the introduction of a sufficient number of viable eggs or gravid females as founders of new populations.

In general, the Integrated Mosquito Management represents the strategic approach for the control of mosquitoes and should be implemented by specialists [92–94]. It should be adopted according to the local conditions as well as to the national/regional regulations and should comprise all appropriate available surveillance and control tools [95]. It comprises usually the following elements:

- Physical control: (i) environmental management (e.g., breeding site reduction) and sanitation (modification of breeding sites e.g., by covering of the containers); (ii) surface layers to chemical and biological obstruct pupal and late larval instars; (iii) reduction of human vector contact e.g., by mosquito windows or bed nets.
- Chemical control: spraying (i) adulticide mainly based on pyrethroids and (ii) larvicides mainly based on biorational chemicals, i.e., with few environmental side effects, like insect growth regulators.
- Biological control: (i) Beside fish and invertebrates (e.g., copepods), microbial control agents, e.g., products based on *Bacillus thuringiensis israelensis* (*Bti*) and *Lysinibacillus sphaericus* are the main biological control tools applied in Europe. (ii) additional products based on *Saccharopolyspora spinosa* or fungi e.g., *Metarhizium anisopliae* and *Beauveria bassiana* as well as the bacterial endosymbiont *Wolbachia* spp. can be used after authority acceptance and registration.
- Genetical control: (i) e.g., the irradiation-based sterile insect techniques (SIT) against tiger mosquito are already successfully used and (ii) genetic engineering can play an important role in future.

The design of the mosquito control strategy has to consider the biology of mosquitoes and their impact on humans. Therefore, e.g., the control strategy for floodwater mosquitoes has to be different from the strategy against urban mosquitoes. With the foundation of the European Mosquito Control Association in 2000 a forum was created for intensive exchange of knowledge in the field of sound mosquito control to solve mosquito problems in Europe.

### Monitoring of the target populations

All control activities should be guided by a well-organized monitoring program to assess the occurrence, abundance and dispersal of the target populations. The monitoring should be implemented by entomologists and should comprise the following techniques: (a) inspections of the breeding sites including larval sampling; (b) employing of ovitraps and adult traps such as Biogents Gravid-*Aedes* Traps or Biogent Sentinel traps; (c) human-landing-collections [1, 93].

Breeding sites can be inspected for mosquito developing stages either by employing a plankton net in larger water containers or pouring the water through a plankton net when small breeding sites are examined [93]. In larger water containers like rainwater barrels the net with a handle should be drawn through the water in a figure of “8” pattern to sample the larvae. By aid of a touch the mosquito developing stages can also recognized in the water column. Nonetheless, in areas with a minor infestation it is difficult to find larvae, different to areas with a dense population where larvae can be easily detected. For transportation to the laboratory, a plastic container with a close-fitting cap filled with 3/4 of water from the site should be used which is carefully marked with the date and location of sampling for determination of the container indices. Third and fourth instar larvae can be identified to species by using appropriate keys [1]. Earlier larval instars should be reared to the 4^th^ instar or even to the adult stage in a mosquito breeder for precise species determination.

Usually ovitraps are the main tool to monitor the presence, phenology and abundance of *Ae. albopictus* and to assess the effect of the control activities (quality control) as well as to estimate the population density based on the number of deposited eggs [96, 97]. The ovitrap usually consist of a dark plastic container with a total volume of about 1.5 L. They are usually filled up to an overflow hole (2/3 of the pot volume) with tap water or hay infusion. A wooden board is added to support oviposition.

In order to prevent the potential development of larvae to imagines a larvicide e.g. granule or tablet formulations based on *Bti* like Vectobac G (activity: 200 international toxic units/mg, Valent BioSciences, Libertyville, USA or Culinex Tab plus, Culinex, Germany) has to be added to the water [92].

Preferable the ovitraps are positioned on shaded places on the ground or hang in a height of max. 1.5 m at places which cannot be easily assessed by animals. The first positioning should be done by a skilled entomologist and the exact position should be registered in an appropriate database with geographical coordinates (e.g., Open Source Geographic Information System) and description of the sampling site for easy assessment. Each ovitrap should have a unique code on the black plastic container and should be always employed on a fixed place during the season.

The density of ovitraps has to be adjusted to the goal of the study. Usually one ovitrap is positioned per two hectares in the infested and in the surrounding areas to determine the spread of the species. The number of ovitraps can also be calculated using the Taylor equation [93].

The wooden boards are usually replaced at a bi-weekly (sometimes weekly or three weekly) interval and the water in the ovitraps has to be refilled. Before refilling the inner walls of the plastic container must be thoroughly cleaned with water and a soft sponge to remove eggs which could be attached to the inner wall of the pot [93]. The collected wooden boards have to be clearly marked at the dry end with a permanent marker to refer to the place of the ovitrap and date of collection. They should be wrapped with paper foil and stored in a plastic bag at room temperature until they are checked by means of a binocular for the presence of eggs.

A skilled person is able to distinguish between eggs of the indigenous species e.g., *Ae. geniculatus* and the exotic species *Ae. albopictus, Ae. koreicus* and *Ae. japonicus*. The results should be validated by hatching some of the eggs and rearing the larvae to the fourth instar for morphological determination or by validation using PCR or MALDI-TOF mass spectrometry [1].

In areas where the sterile insect technique (see below pillar 3) is practiced the sterility of the eggs on wooden boards can be checked by bleaching the exochorion of the eggs by a 10% hydrogen peroxide solution for 48 h and/or disrupting the egg-shell by means of a fine needle to prove existing embryos or unsegmented whitish egg masses.

For quick assessment of the presence of invasive day-time biting *Aedes* species a person can also expose either the whole body or only a part of the body (leg or arm) for a certain time period (some minutes) to collect the approaching mosquitoes by means of an aspirator from the clothes or skin [93]. The species can be determined in the laboratory and the number of biting females per time period can be assessed.

### Control operations

Many control programs employing integrated vector management are based on several pillars, for example pillar (1) community participation; pillar (2) door-to-door activities [92] including the treatment of all breeding sites by trained people with larvicides e.g. *Bti* at appropriate intervals according to the long-lasting effect of the larvicide or a monolayer like aquataine [98]; pillar (3) the integration of the sterile insect technique to possible wipe out remaining *Ae. albopictus* populations. The sterile males are “helpers on wings” by mating with females deriving from cryptic and/or non-accessible breeding sites [92, 93, 95]. The fourth pillar can also include the use of indigenous copepods, *Wolbachia* sp. or genetically modified organisms like OX5034 *Ae. aegypti* females.

In the following the four main pillars of the control strategy against *Ae. albopictus* are described, which mostly also apply to the control of other container-breeding mosquitoes including *Cx. pipiens*. In addition to these four pillars, further measures are described as “Wolbachia—a potential biocontrol agent” and “Epidemiological and resistance risk assessment” are described.

## Pillar I—community participation

Urban areas provide a wide range of water bodies, ranging from flower vases at cemeteries, water barrels, buckets, cans, saucers, water catch basins, bird baths and many more artificial and natural water bodies like tree holes. Mosquito control is therefore particularly successful when people are involved in the frame of community participation [99]. Community participation means that the people are becoming “actors” instead of being “spectators”. It focuses on the increase in public awareness to prevent mosquito breeding as well as to record and report the occurrence of *Ae. albopictus* in the frame of “passive monitoring” as an “early warning system” for the occurrence of invasive species. The programme must enable people to contribute to the solution of their mosquito problem related to their own settlement. “Help through self-help” can be achieved by a comprehensive information campaign. It includes detailed information provided to the public via distribution of leaflets/flyers, press releases, television airtime, web pages and information events, e.g. at schools, in city halls or meetings of gardener associations [93]. Through these activities usually detailed information is given on the characteristics, distribution, and the biology of the Asian tiger mosquito. In addition, easy applying measures should be simple communicated to prevent the proliferation of the mosquito. This includes the elimination of breeding sites, environmental sanitation, e.g., depositing breeding sites like buckets up-site-down that rainwater cannot be collected or the use of totally fitting lids to water containers. Additionally, in heavily infested areas, mosquito nets can be distributed to thoroughly cover water containers as mass breeding sites preventing access for female mosquitoes. Before covering the water barrels larvicides such as *Bti* tablets must be applied to kill the existing larval populations. Thus “help through self-help” facilitates source reduction which has a permanent effect and is highly advantageous from the cost–benefit view. Breeding sites which cannot be removed or sanitated must be treated with larvicides. It is important to keep citizen’s level of motivation high over a long period and to achieve at least 95% of the public. Unfortunately, experiences document that a certain percentage of inhabitants is ignorant and thus limit the success of community participation [92, 93]. Another positive aspect of community participation is that people report suspected mosquitoes in the frame of passive monitoring to the institution/agency/company responsible for the control and thus, immediate surveillance can be organized to prevent further proliferation of the mosquitoes.

### Pillar II—door-to-door control and larviciding in private areas

Due to the lack of professional know-how and the reservation of some people, community participation alone is usually not enough to reach the goal of strong reduction or even elimination of the Asian tiger mosquito populations [92, 100]. Therefore, the implementation of door-to-door activities by trained staff along with the application of long-lasting larvicides e.g. *Bti* treatments in high dosages is highly recommended. Door-to-door is the most powerful tool when control is performed in all properties in regular intervals during the mosquito breeding season (minimum access threshold should be more than 95% of the properties). The staff should be easily identified by letters from the authorities and wearing unique uniforms to support the accessibility of the properties and to destroy concerns of the public. Prior to the actions the public must be informed via the local press that trained staff will visit their properties in the infested area and treat all potential breeding sites with environmentally friendly larvicides which are effective for at least two to three weeks. The necessary time intervals for re-treatments should be assessed in field studies according to the environmental and climate conditions. Door-to-door actions can also be used to distribute flyers to the citizens with thorough information for breeding site elimination and modification. The flyers should contain also contact addresses, telephone numbers, and refer to website pages where people can get additional information and assistance. Additional to flyers mosquito nets for covering mass breeding sites or larvicides for self-help like *Bti* tablets can be distributed that people are able to treat breeding sites e.g., water drums or water catch basins on their private premises. The staff should record all activities including information on the time of treatment, number of permanent breeding sites on a hand-hold computer (mobiles) equipped with a geographic information program. The data base can be programmed that the colour of the treated premises in the display turns to green and change the colour over time till it turns to red (mainly after four weeks). The operator is than aware that he has to re-visit these properties in a certain time. The absence of the owner should also be indicated. It is advisable that during the first round of door-to-door activities all permanent breeding sites/property are mapped as well as such properties which doesn’t contain any breeding sites. The data should be stored in a data base which allow a straightforward planning and targeted control of the properties. Heat maps can be organized which indicate the critical properties and the results of the ovitrap monitoring can also be used to identify critical areas which have to considered for more frequent treatments. The above-mentioned procedure is the optimum way to control *Ae. albopictus* in the private sectors, however, it has been shown that these activities are costly and communities can hardly carry the costs for a long time [97]. Therefore, an alternative is the search for an increased community cooperation by trained inhabitants who are responsible as e.g. “Tiger-mosquito inspectors” for the control in their districts in question. This requires intensive training of the public helpers and guidance by experts.

In the public area all control methods employed in the private sector can be applied but special attention should be taken to water catch basins because they are usually very productive for container-breeding mosquitoes. If sanitation is not possible they have to be treated with larvicides like formulations based on *Bti*. Frequently granule formulations are based on *Bti* or combined products of *Bti* and *Lysinibacillus sphaericus* like VectoMax G. At higher dosages these formulations can also provide a killing effect for at least three weeks.

Beside microbial control agents, insect growth regulators like pyriproxyfen and diflubenzuron as chemicals which cause physiological alterations during the development of insects can be used due to their low acute mammalian toxicology and relative safety to non-target organisms [101]. Insect growth regulators are available as liquid, granular and tablet formulations and registered products are suited to be used in water catch basins. Because of the enormous productivity the treatment of the water catch basins has to carefully conducted in regular intervals by trained staff and checked in the frame of the quality control of the operations [93].

### Pillar III—the sterile insect technique

The final goal of an integrated control program is a significant reduction or ideally the eradication of *Ae. albopictus* populations. In some areas this goal is hardly to achieve where breeding sites are out of reach of community participation and door-to-door control. This applies especially to property owners refusing access as well as to areas with many cryptic breeding sites. In these areas sterile insect technique can be added as the third pillar using e.g., gamma-irradiated sterilizied males. *Aedes albopictus* is ideal for employing sterile insect technique, as the Asian tiger mosquito can easily be mass-reared, has a limited flight range, does not reproduce in enormous numbers within a very short time as the floodwater mosquitoes does, and the breeding sites are well-defined [102]. Preceding the release of sterile males, the natural *Ae. albopictus* population has to be strongly reduced by community participation and door-to-door control [92]. The mass-rearing should be conducted with eggs deriving from the native *Aedes* population of an area where the sterile males will be released to avoid the distribution of another genotype [103–105]. Based on the sexual dimorphism the smaller male pupae can be sieved or sorted out from the female pupae by Fay Morlan glass sorters [106, 107]. Precise sexing is important that the male pupae are not contaminated with female pupae more than an acceptable level of less than 1%. Following the sexing the sorted pupae are sterilized by gamma-radiation at a rate of 1.9 Gy/min for 19 min. resulting in a dosage of 35 Gy which damage the reproductive cell-lines but have no harmful or a limited negative effect to somatic cells to sustain the viability and fitness of the males for flying and mating [108]. The sterile males have to outcompete their wild counterparts resulting in a large majority of wild females laying sterile eggs [108]. In practice the release of about 2000 sterile males/ha on a weekly basis can result in an egg-sterility of more than 80% compared to a natural sterility of less than 5% [92]. The routine release in bi-weekly intervals can result in a reduced sterility of about 60%.

The employment of ovitraps and the final assessment of the sterility by e.g. bleaching the eggs with 10% hydrogen peroxide for 24 up to 48 h allows to assess the effect of the sterile insect technique [92]. The transparency of the egg shell makes it possible to recognize the embryonic structures such as the dark hatching teeth and eyes of the embryos [92] and thus prove the development or non-development of embryos. Taking the operational perspective, over the last ten years the sterile insect technique was tested in different field trials in Montenegro, Germany, Albania, Greece and France [109]. In a recent highly efficient prospective study of a large-scale deployment of the sterile insect technology in Brazil, a > 98% suppression of *Ae. aegypti* live progeny and a 97% reduced incidence of DENV was shown [110]. Thereby, the general effectiveness of the sterile insect technique to reduce the population size of *Ae. albopictus* have be demonstrated. However, there are especially two challenges preventing the usage in routine monitoring. Firstly, regulatory pathways for the release of sterilized mosquitoes are unclear and differ between the countries, and secondly there is a lack of regional factories to produce such mosquitoes.

### Pillar IV—the use of copepods

The application of larvicides results usually only in a sufficient killing effect for a limited number of weeks and therefore a continuous and repeated application of larvicides is required during the mosquito breeding season which is laborious and cost-intensive [92]. Therefore, the search for alternative strategies providing a sustainable long-term control has high priority. Examples of sustainable control is the elimination or sanitation of breeding sites or the use of mosquito nets to cover mass breeding sites like water drums to avoid egg-laying mosquito females. The disadvantage of the majority of larvicides providing only a limited effect can potentially be compensated by the simultaneous inoculation of natural predators to the breeding sites, to feed upon newly hatched larvae as the impact of larvicides ceases. These predators should therefore maintain stable populations within bodies of water, creating a sustainable, long-term vector control [1, 111, 112]. In this context, copepods are considered to be the most efficient invertebrate predators of mosquito larvae and are a promising tool in the control of container-breeding mosquitoes [111]. The use of copepods against *Aedes* mosquitoes was primarily described 1981 by Riviere and Thirel [113]. Most effective are copepods of the largest cyclopoid genera such as *Mega*- or *Macrocyclops* (Cyclopoida: Cyclopidae) which show a positive correlation between their body size and predatory efficiency [114]. These copepods mainly prey on first instar larvae, and to a lesser extent on second instar larvae, as the further developed larval stages exceed the maximum size of potential copepod prey [111, 115]. Furthermore, they show significant differences in their preferences towards different mosquito species [116, 117]. In general, copepod species proved to prey more efficiently upon *Aedes* than *Culex* larvae, indicating their varying prey preferences [111, 118, 119]. It is mandatory that only indigenous copepod species should be considered, since they do not pose any threat towards the local ecosystem and fauna [118, 120].

Promising results using copepods as predators of *Aedes* were achieved in the United States, Asia and South America [119]. However, only a few studies address the use of copepods against *Aedes* species in the UK [114] and Italy [120], while German native copepod species have only been evaluated for their efficacy against the invasive mosquito *Ae. japonicus* in 2019 [121]. In recent laboratory and semi-field test the results reveal a high predation efficiency of *Megacyclops viridis* against first instars of *Ae. albopictus* resulting in a reduction rate of 92.0 ± 12.6% [111]. The copepods did not prey upon stages further developed than the first instars and, in comparison to *Ae. albopictus,* the predation rates on the larvae of *Cx. pipiens* s.l. were significantly lower. The integration of copepods as a promising biocontrol agent to the vector control strategy is therefore highly recommended, especially because of the excellent compatibility of copepods with the use of larvicides like *Bti*. However, the mass rearing of suitable copepods has to be guaranteed. Recently, the copepods as predators of mosquito larvae have been combined with a newly explored mosquitocidal technique applying functionalized nanoparticles representing an emerging tool against virus-transmitting mosquitoes [122–125]. Biologically synthesized toxic silver nanoparticles induce reactive oxygen radicals in the mosquito which target the DNA metabolism and mitochondrial activity of the mosquito larvae. The silver nanoparticles are generated by the endolichenic fungus *Talaromyces funiculosus*. The biomolecule-based nanoparticles in combination with the predatory copepod *Mesocyclops aspericornis* are highly mosquitocidal [123]. Thus, fungal bio-insecticides together with bio-predation by copepods represent a promising green pathway for effective mosquito management processes in future.

### Wolbachia—a potential biocontrol agent

The Gram-negative bacterium *Wolbachia* infects as endosymbiont a wide range of arthropods including a high proportion of insects and nematodes. *Wolbachia* occurs primarily in the sexual organs and manipulates the reproduction of the infected organism in a way that only infected females are able to reproduce [1]. A mechanism which is not fully understood so far but guarantees in the evolutionary process the maternal induced proliferation of the bacterium as endosymbiont. The importance of *Wolbachia* was evident when Yen and Barr [126] found that *Wolbachia* induces cytoplasmatic incompatibility in *Culex* mosquitoes, i.e. the failure of a sperm and egg to produce viable offspring. In case that sperm of a *Wolbachia*-infected *Culex* male fertilizes eggs of a non-infected female it leads to early embryonic death; no viable offspring are produced. Offspring is also not viable when males and females are infected with different strains of *Wolbachia*. Viable offsprings are only produced in a population when both sexes are either uninfected by *Wolbachia* or when an infected female mates with an uninfected male or with an infected male embodying the same *Wolbachia* strain as the female is carrying. Since *Wolbachia* is only transmitted by females, this mechanism favourites the spread of *Wolbachia* and leads to a selection pressure on uninfected females as well as for selected *Wolbachia* strains.

These discoveries have led to new control strategies by introducing *Wolbachia*-infected males into mosquito populations. However, methods of artificial infections of uninfected mosquitoes with *Wolbachia* have been invented, thus opening a new chapter in the control of vectors and arbovirus diseases [127–130]. The *Wolbachia w*Mel strain can reduce the lifespan of adult *Ae. aegypti* and thus reduce the potential for DENV transmission, but these bacteria were not used in Europe so far [129]. The virus-infected vector mosquito doesn’t live long enough to complete the extrinsic incubation period.

The *Wolbachia* release program in Townsville, Australia, led to a 65% reduction in predicted DENV incidence during the release period and over 95% reduction in the 24 months that followed [131]. The release of males of *Ae. albopictus* with a manipulated AR*w*P strain *Wolbachia* induced egg inviability in female mosquitoes which prevents the risk of exotic arbovirus transmission. This population suppression approach is referred as incompatible insect technique [132, 133]. The combination of *Wolbachia*-based incompatible insect technique and radiation-based sterile insect technique can be used for population suppression of *Ae. aegypti*. Using proteomic methods Osario et al. [134] analyzed the seminal proteome of infected males and demonstrated that *Wolbachia* affect the composition of the seminal fluid proteins.

In recent time multiple mechanisms of *Wolbachia*-mediated antiviral activity are detected for instance in *Ae. aegypti* carrying different *Wolbachia* strains [135]. Discovered were changes in RNA processing pathways and upregulation of RNA-binding proteins in the *w*Au *Wolbachia* strain-carrying mosquito, including effects on genes with known antiviral activity. Lipid transport and metabolism proteome changes also differ between strains *w*Au and *w*Mel, respective. In contrast to *w*Mel, the strain *w*Au antiviral activity was not rescued by cyclodextrin treatment. These results suggest that *w*Au could show unique features in its inhibition of arboviruses compared to previously characterized *Wolbachia* strains. Further, *Wolbachia* was shown to interfere with Zika virus replication by hijacking cholesterol metabolism in mosquito cells [136].

However, these bacteria were not used in Europe so far. There is still a huge lack of knowledge regarding the interaction between the bacterium and mosquitoes, which currently prevent the test of field releases of *Wolbachia*-transinfected specimens, e.g., the lack of knowledge on the prevalence of *Wolbachia* on the European-level. In addition, although it can be assumed that the *Wolbachia*-based control approaches might be more accepted than genetically modified mosquitoes, regulatory pathways for the release are largely unexplained.

### Epidemiological and resistance risk assessment

The occurrence of autochthonous transmissions of DENV, ZIKV and CHIKV by *Ae. albopictus* in recent years in Southern Europe is a warning signal and threat underlining the necessity for mosquito surveillance and control activities [38, 41, 43–47]. In case traveller return with proven viremia a careful travel history must be conducted and close cooperation with the health departments is mandatory. Especially in critical areas where *Ae. albopictus* was not yet recorded or in already infested areas, the surveillance must be intensified including the use of adult traps. In case infected mosquito females or even locally acquired infections are detected, immediate control activities must be conducted or intensified. According to the results of the surveillance, the emergency vector control operation has also to include the application of adulticides like pyrethroids when needful [93].

The onset of resistance against control agents can constitute a serious problem especially due to the limited inventory of available products. Therefore, the monitoring of the susceptibility of the target organism as applied product has to be conducted in regular intervals in bioassays e.g. according to the WHO protocols [137]. So far, the use of products based on *Bti* has the advantage that no resistance phenomena against *Bti* could be demonstrated. The rotation of insecticides with different mode of actions can avoid the onset of resistance.

## Conclusions

The emergence of invasive mosquito species, such as t *Ae. albopictus*, and the spread of arboviruses, such as CHIKV and bluetongue virus, have led to an intensification of research and monitoring in Europe. Human mobility dynamics is the primary mode of dispersal on national and regional scales, while transport of goods with plants and tires by ships is the primary mode for continental spread. The risk of further spread of exotic species and mosquito-borne pathogens is expected to increase with ongoing globalization and climate warming. Three invasive mosquito species Asian tiger mosquito, Japanese bush mosquito, and Korean bush mosquito are already established in Europe due to their ability to lay drought-resistant and hibernating eggs. Over the past two decades, there has been an increasing number of outbreaks of mosquito-borne viruses in Europe.

The emergence of exotic mosquitoes and their spread of pathogens, furthermore a transmission contributed by native species warrant the Integrated Mosquito Management for mosquitoes in Europe. Thus, community participation and public awareness by a comprehensive information campaign, should ensure source reduction and successful control of mosquitoes in settlements. Promising control programs represent door-to-door control activities, and integration of sterile insect technique to reduce and possibly wipe out remaining populations (for *Ae. albopictus*) and in addition the use of indigenous copepods, bacteria or genetically modified mosquitoes.

## Data Availability

Not applicable.

## Abbreviations

*Bti*: *Bacillus thuringiensis israelensis*
CHIKV: Chikungunya virus
DENV: Dengue virus
USUV: Usutu virus
WNV: West Nile virus
ZIKV: Zika virus
